# The Placement of Four Short Implants and Full-Arch Early Loading in the Edentulous Patient Suffering from Severe Mandibular Alveolar Ridge Atrophy

**DOI:** 10.1155/2019/1656243

**Published:** 2019-10-22

**Authors:** Yuri Sedov, Oleg Mordanov, Sergei Grigoriev, Anatoly Avanesov, Kamil Khabiev

**Affiliations:** ^1^Department of General and Clinical Dentistry, RUDN University, Medical Institute, Moscow, Russia; ^2^Private Practice, Moscow, Russia; ^3^Private Practice, Cheboksary, Russia

## Abstract

One of the treatments of patients with severe bone atrophy is short implants. It is important to position short implants taking into account the prosthetic loading and right position according to vital anatomical structures. In the presented case report, a seventy-one-year-old female patient underwent the rehabilitation with four short dental implants placed in the anterior mandibula with fully guided surgery to avoid mandibular incisive canal. It solves all the problems in the planning stage when you determine the osteotomy protocol in advance and the prognosis of future restoration according to patient requirements. This clinical case demonstrates the efficiency of patient rehabilitation with the use of short implants in difficult clinical situations.

## 1. Introduction

Dental implants are recommended as a high-quality rehabilitation method for edentulous patients [1, 2]. The survival rates of short dental implants are quite high, on average 91-97% in patients with generalized aggressive periodontitis and 100% in periodontally healthy patients [1]. Not so long ago, there were restrictions on using short implants associated with its stability and prognosis [3]. However, today, there is an evidence-based scientific basis for the effectiveness of the use of short implants in the rehabilitation of patients with severe ridge atrophy [1, 4]. It is often possible not to do additional surgery to augment the tissue volume because of the design of these implants [5, 6]. It is important to note that previously there were age limits, for patients who required implant placement, but at the moment, due to technological improvement of the method, it is not a contraindication to the procedure [7, 8]. Severe bone atrophy makes doctors apply various augmentation methods, but in some clinical situations [9], it is necessary to manage only the available bone [10]. This implies the need for a thorough clinical and radiological examination of the patient in order to be able to place implants and ensure the prosthodontic restoration function [11], especially if the short implants are planned [12].

It has become possible to comply with a specific treatment protocol and improve outcome quality using dental implants even in difficult situations with the development of digital technologies in dentistry. Also, it is possible to reduce the number of possible complications and achieve favorable clinical outcomes through such approach [13].

The aim of this clinical report is to demonstrate the rehabilitation of the patient using four immediate short implants after extraction of all teeth and early prosthetics.

## 2. Case Report

A seventy-one-year-old female patient came to the dental practice because of poor denture fixation on the mandible. Previously, the denture was corrected and adjusted, but it was ineffective. The maxilla also had a complete removable prosthesis, which was suited to the patient. The patient noted the occurrence of pain in the projection of the mental foramens during the closing of the jaws with the prostheses.

**Figure 1:**
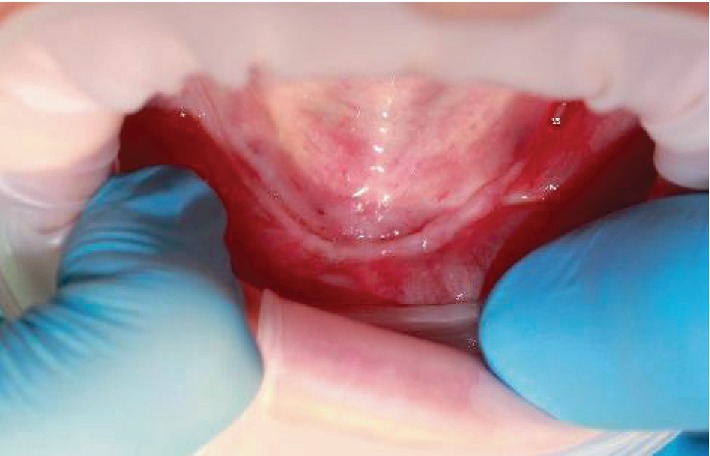
Oral cavity examination. Severe atrophy of the alveolar mandible.

The absence of the border between vestibule and oral cavity proper was noted during the clinical examination (Figure 1). There was a marked deficiency of the mandibular alveolar ridge.

**Figure 2:**
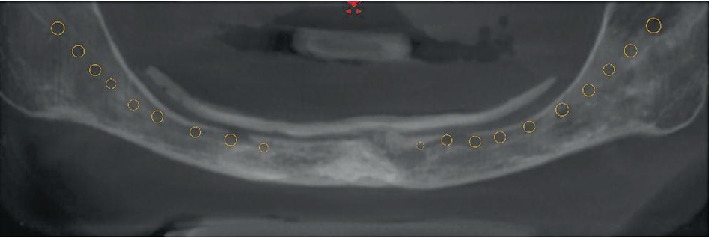
Panoramic view of the CBCT scan. The mandibular canal is traced. The opening of a neurovascular bundle under soft tissue is noted.

The CBCT scan showed a close position of the mandibular canal with the opening of the inferior alveolar nerve from the mental foramens under soft tissues, which was confirmed by the patient's complaints (Figure 2). She was offered treatment options with dental implants and bone augmentation. The patient refused bone augmentation and chose the option with the short implant placement in the anterior mandible. A strict treatment plan was individually prescribed, which included the following points:

1. Impressions and cast models fabrication
2. Making a radiopaque mould for CBCT
3. CBCT examination along with the individual radiopaque mould
4. Scanning of cast models and the radiopaque mould
5. Comparison of scan and tomography files for complete planning
6. Creating a surgical guide
7. Fully guided dental implant placement
8. Prosthesis fabrication

After the patient's consent with this option, the first three stages were performed. The models were cast, and the radiopaque mould was made of polymer and base wax to record the occlusion height. At last, a layer of the radiopaque basis of the mould was visible. Then, the models of the upper and lower jaws were scanned separately and together with a mould to create an occlusal file for further virtual wax-up. Also, the mould and the mould with the models of the mandible were also scanned separately. The last two scans are needed for comparison with CBCT. Thus, five scans were obtained.

The R2Gate (Megagen, South Korea) software made a comparison of computed tomography data presented in the ∗.dcm and scans in the ∗.stl. The correlation between mandibular scan and its CBCT scan was matched with the help of the radiopaque mould in the CBCT scan and the 3D scans of this mould.

**Figure 3:**
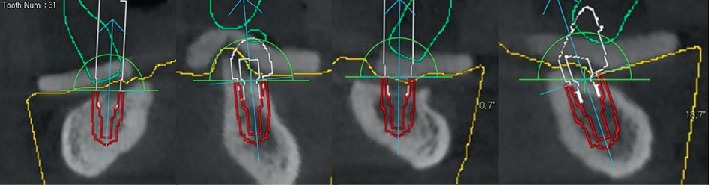
The CBCT scan. Coronal mandibular view. Four implants were placed in the 3.2, 3.4, 4.1, and 4.3 teeth regions. Distal implants are tilted at 29 degrees.

Further, the available bone in the anterior mandible was assessed. The location of the mandibular incisal canal in the implant site was noted, and the patient was informed, including possible complications after surgery. Four implants are virtually placed, two of which are positioned tilted at 29 degrees due to the lack of available bone (Figure 3).

The final correlation between the implants and the virtual wax-up made it possible to produce a splinted prosthesis, since the scan abutments were relatively parallel to each other.

Next, the design of the surgical guide was developed and printed with a print thickness of 50 microns. Separately, a protocol of recommended drilling was developed for obtaining favorable primary implant stability. It was based on the size of the platform, apex, and type of bone density based on the classification by Lekholm and Zarb [12]. Additionally, a silicone index was made for fixing the guide with the opposite jaw at the time of the pin placement for guide stabilization. Before the surgery, the patient was prescribed an antibiotic (lincomycin 500 mg).

## 3. Surgery Protocol

**Figure 4:**
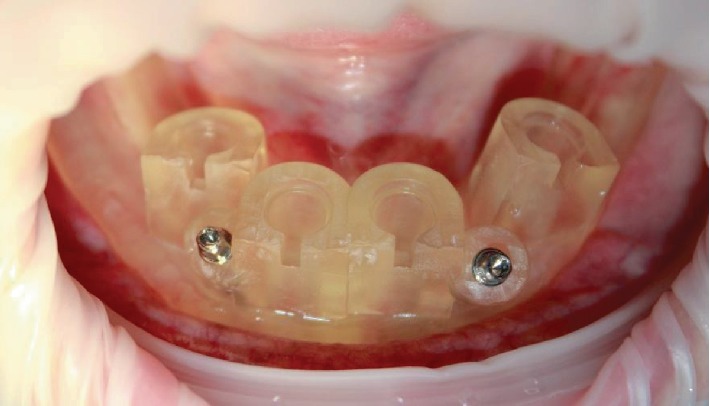
Fixed surgical guide.

The general condition is satisfactory; there were no complaints. Antiseptic was performed on the oral cavity with a solution of chlorhexidine digluconate 0.05%, then the local anesthesia was carried out. A surgical guide is placed on the lower jaw, then a silicone index is positioned; after that, the patient squeezed the jaws to fix the position of the guide. Fixing pins were placed and the silicone index was removed (Figure 4).

The first drilling with a special mucotomy-type transgingival drill was performed with the guide to remove the soft tissue. As surgical guides limit irrigation during the drilling, sequential drilling was carried out at a drilling speed of 350 rpm, according to the previously developed protocol. The drills had a cylinder in the apical section with a diameter that was equal to the walls of the guide. The drill's free movement was excluded due to the sequential drilling from a short drill to the required height. Four implants (4.1 region—4∗7 *mm*, 3.2 region—4∗7 *mm*, 4.3 region—4∗7 *mm* with 29-degree angulation, 3.4 region—3.5∗7 *mm* with 29-degree angulation; AnyOne, Megagen, South Korea) were placed through the guide using a special implant driver for precise height adjustment.

**Figure 5:**
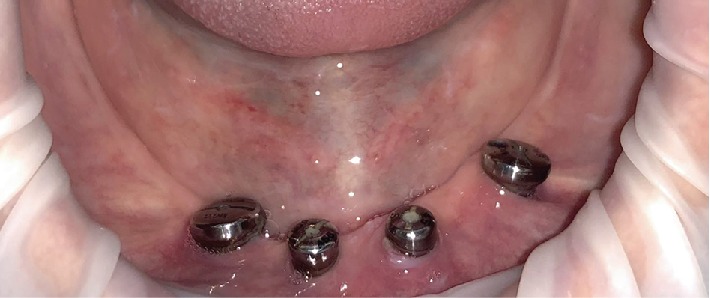
Healing abutments in situ.

The final torque on the implants was 40 *N*∗*cm*. However, due to the fact that a number of implants were placed in the lower incisal canal, it was decided to install only the healing abutments (Figure 5). Closure was not necessary due to the work with the mucotome.

**Figure 6:**
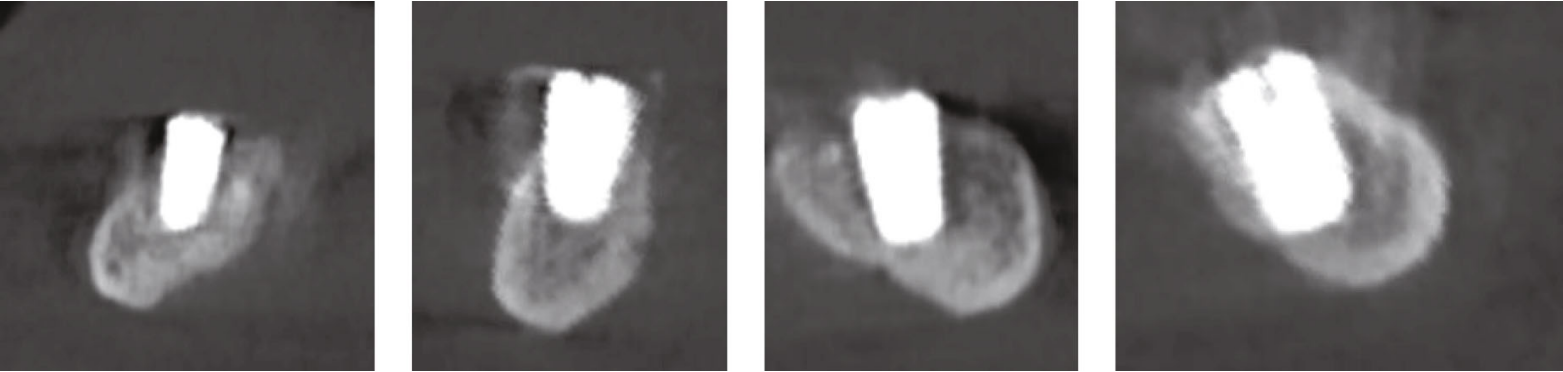
СBCT scan with implants in situ.

An antiseptic with chlorhexidine digluconate 0.05% was performed. The radiographic control was provided (Figure 6). Prescriptions including antibiotic prophylaxis were given (lincomycin 500 mg, 1 capsule bis in die for 5 days), and antihistamine and nonsteroidal anti-inflammatory drugs were also prescribed. The existing prosthesis is rebased according to the healing abutments.

The general condition was satisfactory in the next follow-ups; the postoperative region was without inflammatory changes.

**Figure 7:**
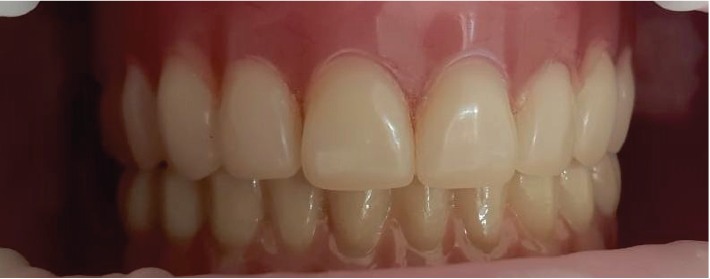
Removable maxillary- and implant-supported mandibular prosthesis in situ.

A new removable maxillary prosthesis was agreed upon with the patient. Due to pronounced financial constraints, she had to deny the usual prosthesis on multiunit abutments. As implants were placed with a surgical guide, and when planning they were all derived as parallel as possible, transfer checks on titanium bases without a hexagon were previously manufactured. Despite the angulation of the implants, after the try-in of on-transfer checks in the oral cavity, a decision was made about the possibility of manufacturing an all-zirconium prosthesis on these titanium bases. The durability of this design provides contact with a removable denture of the maxilla, so there is no overpressure (Figure 7).

The whole zirconium frame was virtually designed after the determination and analysis of all the nuances on the previously obtained STL file with the position of the scan abutments and CAM fabrication.

**Figure 8:**
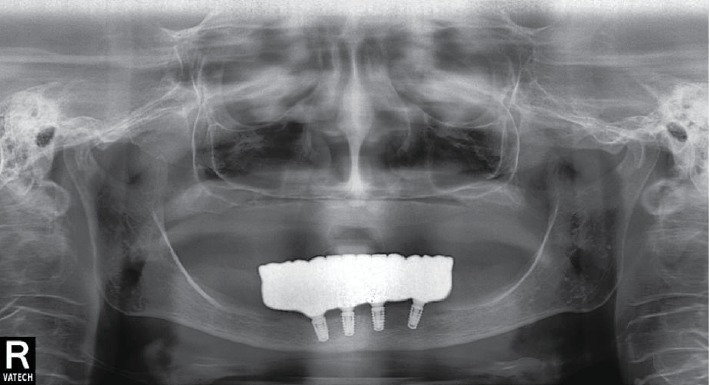
Panoramic view. Radiographic follow-up a year after surgery.

The patient follows the recommendations for hygiene and undergoes follow-ups every six months (Figure 8).

## 4. Discussion and Conclusions

This case report shows the compilation of using short implants in an old patient with fully guided surgery. Short implants have proven their effectiveness with both immediate and delayed loads [4]. In patients with a complex clinical view and the impossibility of restoring bone volume, the use of short implants is a good alternative [1, 4]. The use of short dental implants decreases the need for complicated surgical bone augmentation procedures, which reduces complications, costs, treatment time, and morbidity [14]. Also, the treatment plan with short implants can also be used in age-related patients [13].

There are three types of patterns used for edentulous arches: those designed for freehand drilling, those designed for semiguided controlled directional drilling, and those designed for fully guided controlled directional drilling. Fully guided surgical templates are used when precise control of angulation, location in the arch, and apicocoronal depth are critical to the success of the definitive prosthesis [15]. Fully guided surgery has been proven to be a reliable and accurate method that reduces the damage to the alveolar nerve, sinus perforation, fenestration, etc. [16]. This guide provided not only precise implant placement but also flapless surgery whose advantage is the minimal surgical procedure that supports the preservation of the blood circulation in the soft tissues, which may affect the soft-tissue architecture and reduce bone loss [17, 18].

All these factors combined into a single implant treatment provided the successful implant placement in an old patient without complications. Though the possibilities of short implants are limited, they require further study from the point of view of long-term survival in difficult clinical situations in such patients with such methods described in this article.
